# Testing the Efficacy of Cabozantinib (XL-184) to Treat Glioblastoma

**DOI:** 10.51894/001c.144110

**Published:** 2025-09-30

**Authors:** Monish Moyal

**Affiliations:** 1 College of Osteopathic Medicine Michigan State University

## 32

Glioblastoma (GBM) is a highly aggressive brain malignancy with limited therapeutic advancements. This study evaluates the efficacy of cabozantinib (XL184), a multi-kinase inhibitor targeting VEGFR2, MET, and AXL, in GBM cancer stem cells (CSCs) and patient-derived xenograft (PDX) models. Using nine genomically diverse GBM CSC lines, we observed a wide range of XL184 sensitivity (IC50: 2μM - 34 μM) independent of genomic alterations. Reverse Phase Protein Array (RPPA) analysis revealed XL184-mediated inhibition of phospho-VEGFR2, AKT, and ERK, while sub-lethal doses upregulated phospho-MET and STAT3, suggesting adaptive resistance mechanisms.

In vivo, orthotopic xenografts treated with XL184 demonstrated differential responses. HF2587 tumors exhibited significant growth reduction and improved survival with XL184 monotherapy (p<0.01). In contrast, XL184 alone was ineffective in HF2303 tumors; however, combination with temozolomide (TMZ) enhanced tumor regression and extended survival (p<0.05). CD-31 based immunohistochemical analysis of tumor sections, through Keyence microscopy, indicated XL184’s has significant reduction in vessel density in some groups, thereby indicating an impact on angiogenesis and tumor microenvironment modulation.

These findings highlight the heterogeneity of XL184 response in GBM and the potential for combination strategies to overcome resistance. Insights into CSC signaling and tumor-specific dependencies provide a foundation for biomarker-driven, personalized therapeutic approaches in GBM.

